# A culturomics-inspired multi-medium workflow for soil fungal isolation and ITS metabarcoding comparison

**DOI:** 10.1016/j.mex.2026.104179

**Published:** 2026-09-27

**Authors:** Charbel Elias, Aïcha Hamieh, Stéphane Ranque, Laure Malleret

**Affiliations:** a Aix Marseille University, SSA, AP-HM, UMR D, Risques Infectieux, Microorganismes Emergents (RITMES), Marseille, France; b Aix Marseille University – CNRS, Laboratoire Chimie Environnement (LCE) , UMR 7376, Marseille, France; c Institut Hospitalo-Universitaire Méditerranée Infection (IHU), Aix Marseille Université, Marseille, France; d Aix Marseille University – APHM, MEPHI, Marseille, France

**Keywords:** Soil fungi, Fungal isolation, Culturomics-inspired workflow, MALDI-TOF MS, Multilocus sequencing, ITS metabarcoding, Environmental mycology, Fungal taxonomic identification

## Abstract

This method article describes a reproducible workflow for recovering, identifying, and comparing soil-associated fungi using complementary culture-dependent and sequence-based approaches. The workflow was designed for environmental mycology studies in which fungal DNA detection needs to be complemented by the recovery of living isolates. It combines standardized soil suspension processing, parallel plating on five culture media, daily morphotype selection and purification, MALDI-TOF MS screening, targeted multilocus sequencing when required, and comparison with ITS1/ITS2 metabarcoding profiles. In its application to soil samples, the workflow recovered a diverse viable isolate collection, supporting its usefulness for expanding fungal recovery, improving lower-level taxonomic anchoring, enriching identification resources, and distinguishing culture-recovered from sequence-detected fungal fractions.

• Five culture media are used in parallel to broaden the spectrum of viable soil fungi recovered under standardized incubation conditions.
• Recovered isolates are identified using MALDI-TOF MS and targeted multilocus sequencing when necessary.
• Culture-recovered taxa are compared with ITS1/ITS2 metabarcoding profiles to distinguish shared and method-specific fungal fractions.

## Graphical abstract

Graphical abstract. Overview of the proposed methodological workflow for soil fungal diversity profiling, from soil sampling and culture-dependent isolation on multiple media to isolate identification, ITS1/ITS2 metabarcoding, and comparative integration of culture-based and sequence-based datasets.

## Specifications table

**Table:** 

**Subject area**	Agricultural and Biological Sciences
**More specific subject area**	Environmental mycology; soil fungal isolation; fungal metabarcoding; microbiological identification workflows
**Name of your method**	Culturomics-inspired multi-medium workflow for soil fungal isolation, isolate identification, and ITS metabarcoding comparison
**Name and reference of original method**	The workflow adapts the general culturomics principle of diversifying culture conditions to broaden microbial recovery [[1], [2], [3]], incorporates FastFung agar as an enriched medium originally developed for fastidious clinical fungi [4], and combines MALDI-TOF MS fungal identification [5,6] with ITS-based sequence analysis and metabarcoding tools [7,8], implemented within QIIME2 [9].
**Resource availability**	Raw ITS metabarcoding data are available in NCBI SRA under accession PRJNA1118208. Sanger sequences from culture-recovered isolates are deposited in the NCBI GenBank nucleotide database under accession numbers PPP851664–PP851799 (ITS), PP892421–PP892523 (β-tubulin), PP869633–PP869652 (TEF1-α), PP860821–PP860826 (D1/D2 LSU), and PP869628–PP869632 (RPB2); isolate-specific accession information is provided in Supplementary Table 6. Supplementary tables from the related research article include sample descriptions, primer sets, MALDI-TOF MS/molecular identification results, ITS1/ITS2 taxonomy tables, and GenBank accession numbers. Software used includes QIIME2 2019.2, DADA2, VSEARCH, MicrobiomeAnalyst 2.0, MEGA11, FastTree, iTOL, and GraphPad Prism. MALDI-TOF MS analyses used the Bruker Microflex LT/MBT workflow with commercial and in-house spectra databases.

## Background

Soil fungal diversity is commonly investigated using ITS metabarcoding because this approach can detect a broad range of fungal DNA signatures from complex environmental samples. However, sequence-based detection does not recover living organisms and may provide limited resolution for some fungal groups, especially at lower taxonomic levels. Conversely, culture-dependent isolation provides viable fungi that can be preserved, re-identified, phenotypically characterized, and tested in downstream functional assays, but culture recovery is strongly influenced by medium composition, incubation parameters, and competition among colonies.

The method described here was developed to bridge these two complementary perspectives. It applies a streamlined culturomics-inspired strategy to soil fungal isolation by using five culture media in parallel under standardized conditions, followed by MALDI-TOF MS identification and multilocus sequencing when necessary. MALDI-TOF MS is a key component of this workflow because it enables rapid preliminary identification of large numbers of purified colonies, which is particularly important in a multi-medium culture strategy where many morphologically distinct isolates may be recovered and would otherwise require time-consuming molecular identification. The same soil sample set is also analyzed by ITS1/ITS2 metabarcoding, allowing culture-recovered taxa to be compared with sequence-detected taxa. The workflow is therefore designed for studies that aim to generate viable fungal resources while also evaluating how culture-dependent recovery overlaps with, and differs from, metabarcoding-based detection.

The method is particularly useful when researchers want to evaluate fungal taxon recovery and infer soil fungal community structure. It provides practical information about medium-dependent recovery patterns, identifies fungi that can be recovered as viable isolates, and highlights the added value of isolate-based taxonomic anchoring for fungal groups that may not be reliably resolved using short ITS amplicons alone.

## Method details

### Overview of the workflow

The workflow consists of eight linked stages: (1) soil homogenization and storage, (2) preparation of soil suspension and serial dilutions, (3) parallel plating on five fungal culture media, (4) daily colony monitoring and purification of distinct morphotypes, (5) MALDI-TOF MS screening, (6) targeted multilocus sequencing of isolates requiring confirmation, (7) ITS1/ITS2 metabarcoding and bioinformatic processing, and (8) comparative analysis of culture-recovered and sequence-detected taxa. The complete culture-dependent and sequence-based workflow is summarized in Fig. 1.

**Fig. 1:**
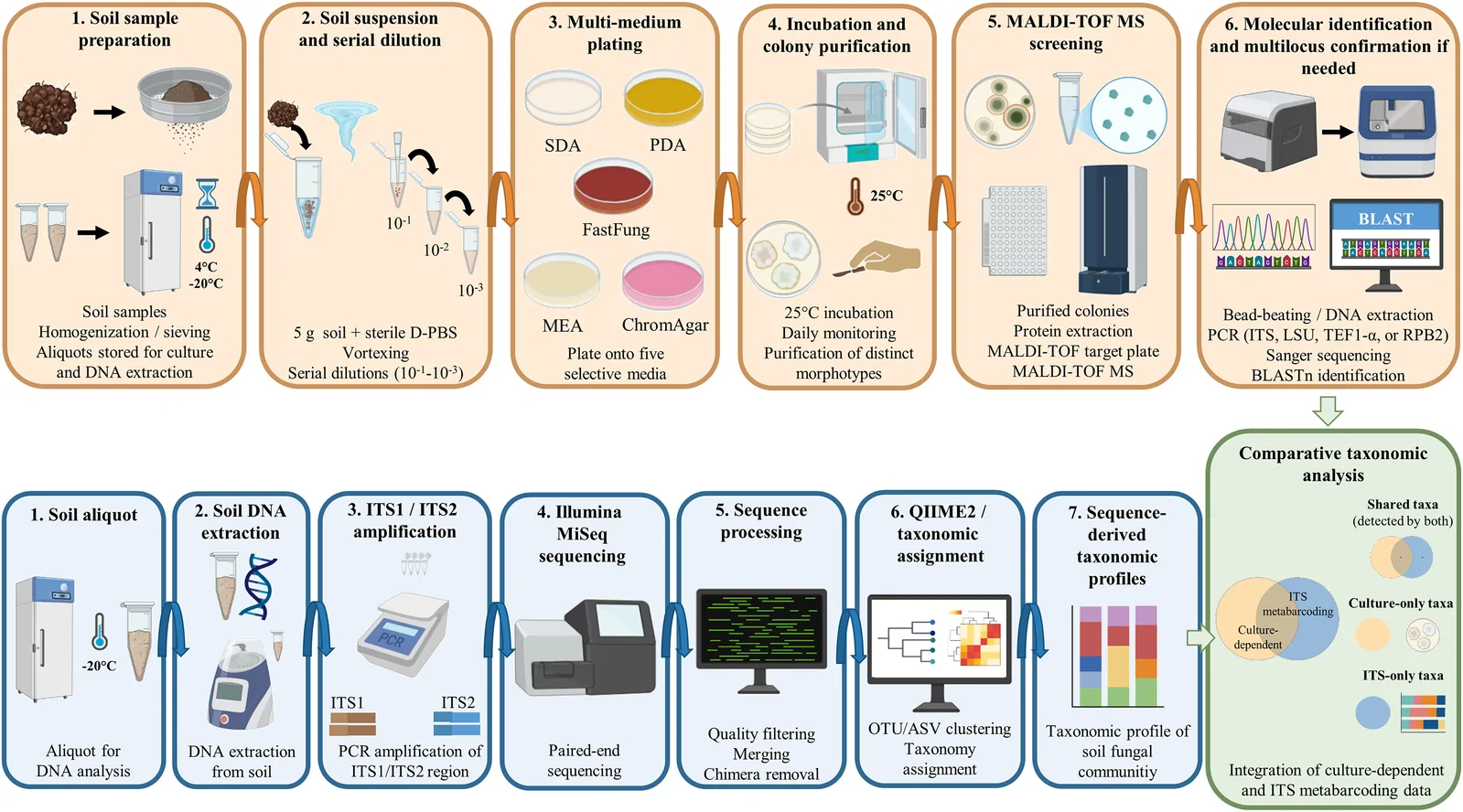
Overview of the culture-dependent and ITS metabarcoding workflow used for soil fungal diversity profiling. The culture-dependent branch includes soil sample preparation, soil suspension and serial dilution, multi-medium plating, incubation and colony purification, MALDI-TOF MS screening, and molecular confirmation when required. The sequence-based branch includes soil DNA extraction, ITS1/ITS2 amplification, Illumina MiSeq sequencing, bioinformatic processing, taxonomic assignment, and comparative integration of culture-recovered and sequence-detected taxa.

#### Soil sample preparation

Collect soil samples from the depth or soil horizon relevant to the study design, and record the sampling depth as part of the sample metadata. After air drying, homogenize each sample. In the related study, the surface litter layer was removed before sampling, and soil was collected primarily from approximately 10–20 cm depth. Sieve the soil through a 2-mm mesh to remove large debris and improve consistency. Following homogenization, soil aliquots were stored at 4 °C for culture-dependent isolation, while parallel aliquots were stored at −20 °C until subsequent DNA extraction. In the related research article, approximately 1 kg of each soil sample was processed, and 19 soil samples from southeastern France were analyzed by ITS metabarcoding, with 14 of these also subjected to culture-dependent isolation.

#### Soil suspension and dilution

For culture-dependent isolation, prepare a single suspension per soil sample by suspending approximately 5 g of soil in 15 mL sterile Dulbecco’s phosphate-buffered saline (DPBS). Vortex the suspension to dislodge fungal propagules. Prepare serial dilutions from 10^−1^ to 10^−4^ in sterile DPBS. The dilution series reduces microbial load and limits immediate overgrowth by the fastest-growing colonies.

#### Multi-medium plating

Plate 300 µL of each dilution once onto each of five 90-mm culture plates containing different fungal culture media: Sabouraud Dextrose Agar supplemented with gentamicin (50 mg/L) and chloramphenicol (50 mg/L) (SGC2; bioMérieux); Potato Dextrose Agar (PDA; NutriSelect® Basic, P2182, MilliporeSigma) supplemented with chloramphenicol (50 mg/L); modified Malt Extract Agar (MEA), containing malt extract (20 g/L), peptone (1 g/L), glucose (20 g/L), and agar (20 g/L), adjusted to pH 5.6 ± 0.2 and supplemented with chloramphenicol (50 mg/L); BBL™ CHROMagar™ Candida Medium (BD), supplied as commercially prepared plates and used as provided; and FastFung agar, prepared as described by Bittar et al. [4]. The term ‘modified MEA’ refers here to this enriched formulation, which includes peptone and glucose in addition to malt extract and agar.

These media were selected to diversify nutrient composition and selective properties while keeping incubation conditions standardized.

Include uninoculated sterility-control plates for each medium batch and incubate them under the same conditions as inoculated plates. This control step is important to distinguish genuine soil-derived fungal recovery from medium or handling contamination.

#### Incubation and colony purification

Incubate plates aerobically at 25 °C for up to 7 days. Examine plates every 24 h. Newly visible colonies are assessed for morphological uniqueness based on colony colour, texture, margin, growth form, and other macroscopic features. Distinct morphotypes are subcultured onto the corresponding isolation medium until pure isolates are obtained. A relatively short incubation period is used to limit overgrowth by rapidly expanding colonies and preserve comparability among samples and media.

#### MALDI-TOF MS isolate identification

Subject purified fungal colonies to protein extraction prior to MALDI-TOF MS identification. In the related research workflow, colonies were suspended in a microcentrifuge tube containing 1 mL of 70% ethanol. Centrifuge the tube at 13,000 rpm for 5 min for yeasts or 10 min for filamentous fungi. Discard the supernatant, centrifuge again for 2 min at 13,000 rpm, carefully remove the remaining liquid with a pipette, and allow the pellet to dry.

Under a chemical hood, add 10 µL of 70% formic acid to the dried pellet and homogenize thoroughly by pipetting. Incubate for 5 min with the tube closed. Then add 10 µL of 100% acetonitrile, homogenize again by pipetting, and incubate for another 5 min with the tube closed. Centrifuge the tube for 2 min at 13,000 rpm and use the resulting supernatant for MALDI-TOF MS spotting.

Deposit 1 µL of the supernatant onto the MALDI target plate. In the related workflow, one spot was used for yeasts and four spots were used for filamentous fungi to increase the chance of obtaining interpretable spectra. Deposit 1 µL of calibrant on the target plate and allow all spots to dry at room temperature. Then overlay each sample spot with 1 µL of HCCA matrix, allow the plate to dry again at room temperature, and analyse the plate using a Bruker Microflex LT MALDI-TOF MS instrument and the MALDI Biotyper system.

Use both a commercial MALDI-TOF MS reference library and an in-house fungal spectra database where available. In the related study, identifications were accepted when the Biotyper log(score) value was ≥1.95, according to the previously established predefined conservative criterion routinely applied for filamentous fungal identification within our institute and mycology laboratory. Despite this difference in spotting strategy, the same conservative Biotyper log(score) acceptance threshold of ≥1.95 was applied to both yeast and filamentous fungal isolates. This conservative institutional criterion is consistent with previous evaluations of MALDI-TOF MS identification thresholds for filamentous fungi [6]. Isolates with no reliable MALDI-TOF MS identification, borderline scores, or taxonomic complexity are moved to molecular identification. The MALDI-TOF MS screening and molecular confirmation decision workflow is summarized in Fig. 2.

**Fig. 2:**
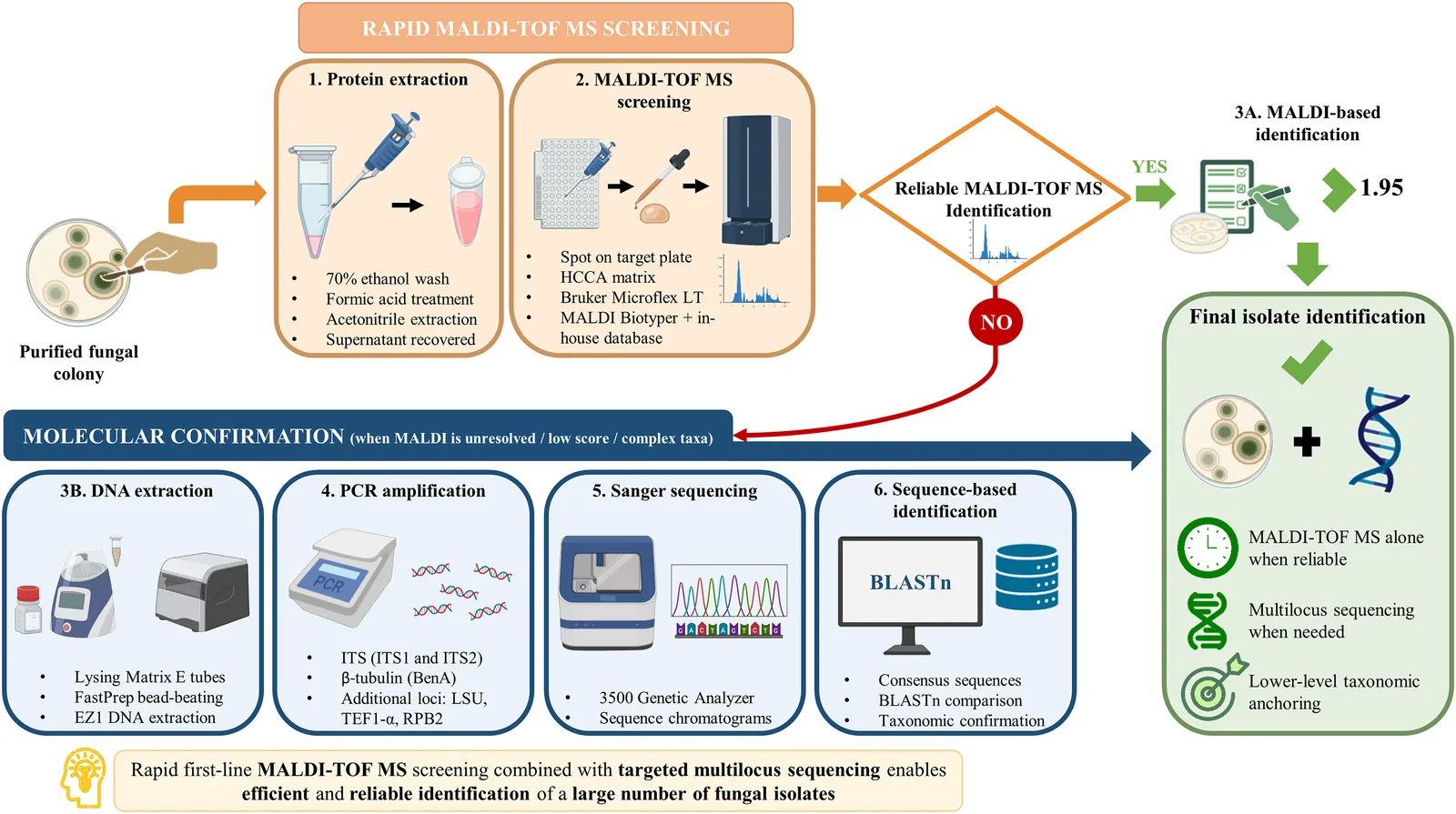
Rapid MALDI-TOF MS screening and targeted multilocus sequencing workflow for fungal isolate identification. Purified fungal colonies are first subjected to protein extraction and MALDI-TOF MS screening. Isolates with reliable MALDI-TOF MS identification are retained directly, whereas unresolved, low-score, or taxonomically complex isolates are processed by DNA extraction, PCR amplification, Sanger sequencing, and BLASTn-based molecular confirmation.

#### Molecular identification and multilocus confirmation

For isolates requiring molecular identification, perform DNA extraction and Sanger sequencing as a second-line or confirmatory identification step. This step should be applied to isolates that remain unidentified by MALDI-TOF MS, isolates with borderline or low-confidence MALDI-TOF MS scores, and isolates belonging to genera requiring higher taxonomic resolution because of species complexes or limited reference database coverage.

Begin with a mechanical lysis step to disrupt the fungal cell wall and release intracellular material. Transfer fungal biomass from a purified colony into a Lysing Matrix E tube (MP Biomedicals, USA) containing ceramic and glass beads. Add 700 µL of NUCLISENS® easyMAG® lysis buffer (bioMérieux, France) to the tube. Perform mechanical lysis using a FastPrep-24 5 G bead-beating system (MP Biomedicals, USA). After lysis, centrifuge the tube and collect 200 µL of supernatant for DNA extraction. Extract total DNA using a BioRobot EZ1 automated extraction system (Qiagen, Les Ulis, France) with the EZ1 DNA Tissue Kit (Qiagen, France), following the manufacturer’s instructions.

Select molecular markers according to the preliminary taxonomic assignment and the level of resolution required. MALDI-TOF MS provides the initial identification step; when no reliable identification is obtained, ITS sequencing is used as a first-line broad fungal barcode. The preliminary identification obtained from MALDI-TOF MS and/or ITS sequencing then guides the selection of additional, more discriminating barcode regions according to the fungal genus. In the present workflow, β-tubulin (β-tub2) was used particularly for Aspergillus, Penicillium and Talaromyces, partial translation elongation factor 1-alpha (TEF1-α) for Fusarium, and partial RNA polymerase II subunit 2 (RPB2) when additional taxonomic resolution was required. The D1/D2 domain of the large ribosomal subunit (LSU/28S) was also used for selected isolates. Primer sequences for all loci are provided in Supplementary Table 2.

For PCR amplification, add 5 µL of extracted DNA to 20 µL of PCR mix, for a final reaction volume of 25 µL. Prepare the PCR mix using 12.5 µL of AmpliTaq Gold™ 360 Master Mix (Applied Biosystems), 6 µL of sterile DNase/RNase-free water, and 0.75 µL of each forward and reverse primer. Perform PCR amplification using the following cycling conditions: initial denaturation at 95 °C for 15 min; 39 cycles of denaturation at 95 °C for 1 min, annealing at 56 °C for 30 s, and extension at 72 °C for 1 min; followed by a final extension at 72 °C for 5 min.

Sequence the amplified products by Sanger sequencing using a 3500 Genetic Analyzer (Applied Biosystems, Villebon-sur-Yvette, France). Assemble, inspect, and manually correct raw sequence reads using ChromasPro version 1.34 (Technelysium Pty Ltd, South Brisbane, Australia). Compare the resulting consensus sequences against the NCBI rRNA/ITS and standard nucleotide databases using BLASTn. A minimum BLASTn sequence similarity of 99% was used as the operational criterion for considering a species-level identification, with best-matching sequences derived from type material or well-characterized reference strains prioritized whenever available. Species-level identification was not based on percentage identity alone. When several species or strains showed similarly high sequence similarity, or when the primary marker did not provide sufficient discriminatory power, BLASTn results were interpreted together with additional loci selected according to the fungal genus. Concordant results from these genus-appropriate secondary markers were used to resolve the identification where possible; otherwise, the isolate was retained at the genus or species-complex level.

This multilocus confirmation step is particularly important for species-rich or taxonomically complex genera such as *Aspergillus, Penicillium, Fusarium, Trichoderma*, and *Talaromyces*, for which ITS alone may not reliably distinguish closely related species. In the workflow, MALDI-TOF MS therefore provides rapid first-line screening of purified isolates, while targeted molecular identification provides taxonomic confirmation and finer resolution when MALDI-TOF MS or ITS alone is insufficient.

#### ITS1/ITS2 metabarcoding and sequence processing

Extract total DNA from 500 mg of each homogenized soil sample using the FastDNA® Spin Kit for Soil (MP Biomedicals, Solon, OH, USA), following the manufacturer’s instructions. Amplify ITS1 and ITS2 separately in order to compare the taxonomic profiles generated by two commonly used fungal ITS markers. For ITS1, use the ITS1-F primer (5′-CTTGGTCATTTAGAGGAAGTAA-3′) together with ITS2 (5′-GCTGCGTTCTTCATCGATGC-3′). For ITS2, use ITS3 (5′-GCATCGATGAAGAACGCAGC-3′) together with ITS4 (5′-TCCTCCGCTTATTGATATGC-3′). Use Illumina-compatible sequencing adapters during amplification or subsequent library preparation, depending on the sequencing platform workflow. The ITS1/ITS2 metabarcoding and comparative taxonomic integration workflow is summarized in Fig. 3.

**Fig. 3:**
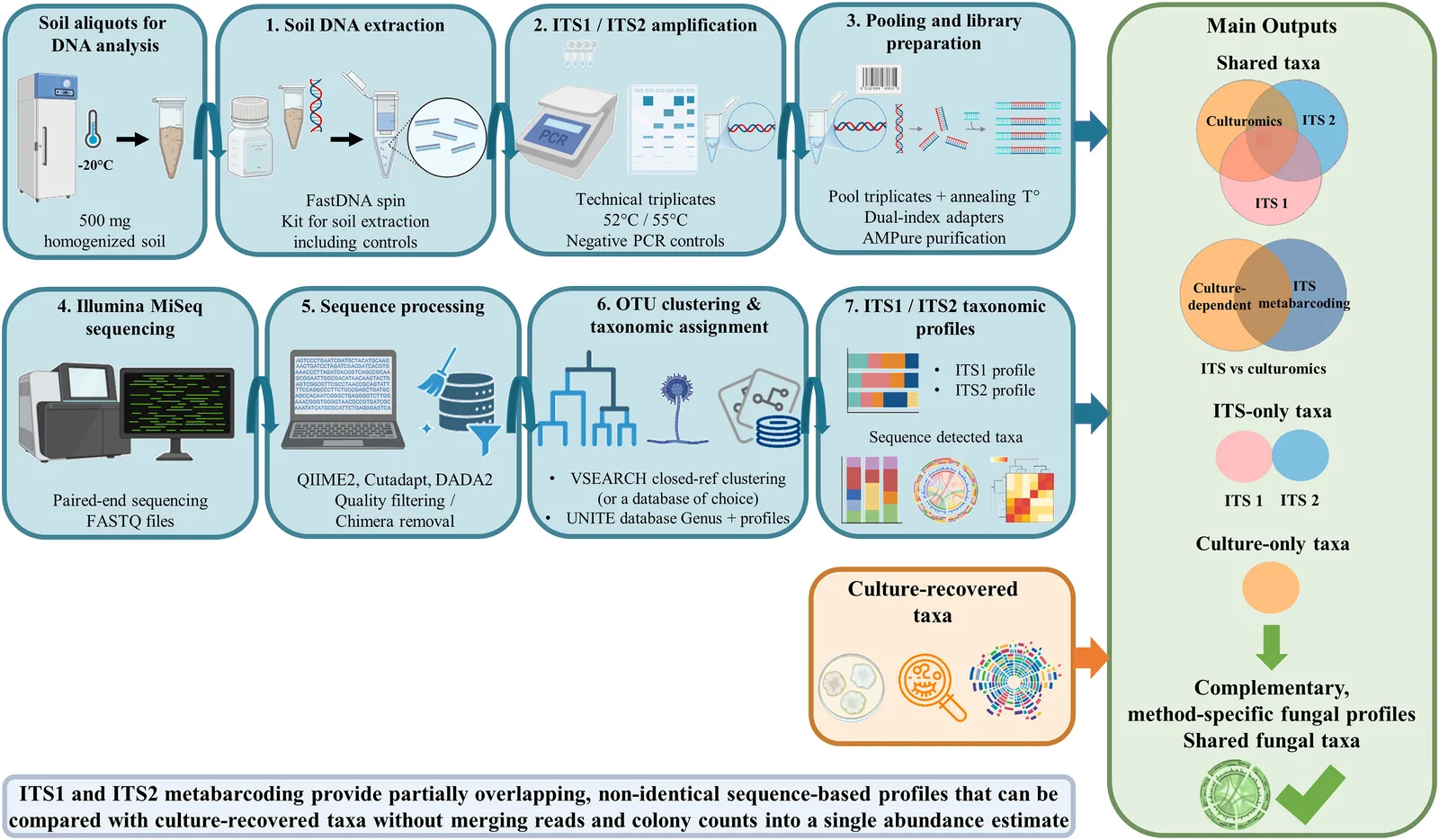
ITS1/ITS2 metabarcoding workflow and comparative integration with culture-recovered fungal taxa. Soil DNA is extracted, amplified using ITS1 and ITS2 markers, sequenced by Illumina MiSeq, processed using QIIME2-based bioinformatic steps, and taxonomically assigned. The resulting ITS1/ITS2 profiles are compared with culture-recovered taxa using a presence/absence-based framework to distinguish shared, ITS-only, and culture-only fungal fractions.

For each soil sample and each marker, perform PCR reactions in technical triplicate and at two annealing temperatures, 52 °C and 55 °C, to increase amplification success across diverse fungal templates. Prepare each 25 µL PCR reaction using 12.5 µL of AmpliTaq Gold™ 360 Master Mix, 6 µL of sterile DNase/RNase-free water, 0.75 µL of each forward and reverse primer, and 5 µL of template DNA. Perform PCR amplification using the following cycling program: initial denaturation at 95 °C for 10 min; 40 cycles of denaturation at 95 °C for 5 s, annealing at 52 °C or 55 °C for 30 s, and extension at 72 °C for 1 min; followed by a final extension at 72 °C for 5 min.

Pool amplicons obtained from the technical replicates and annealing temperatures for each sample and marker before library preparation. Add dual-index sequencing adapters by indexing PCR according to the Illumina-compatible workflow used by the sequencing platform. Purify the amplified and indexed libraries using AMPure beads (Beckman Coulter Inc., Fullerton, CA, USA). Pool the barcoded libraries together with other multiplexed libraries when applicable. Quantify the pooled library using a high-sensitivity Qubit assay (Life Technologies, Carlsbad, CA, USA), dilute the pool to 8 pM, and load it onto an Illumina MiSeq™ sequencing platform for paired-end sequencing with dual-index reads. After sequencing, generate paired-end FASTQ files corresponding to forward (R1) and reverse (R2) reads. Process extraction and PCR negative controls alongside soil samples to monitor possible reagent or laboratory contamination.

Process sequence data using QIIME2. Demultiplex reads, remove primers/adapters using Cutadapt, and perform quality filtering, denoising, chimera removal, and amplicon sequence variant (ASV) inference using the DADA2 plug-in. Trim and truncate reads according to quality score profiles; in the related workflow, reads were truncated to avoid low-quality regions after approximately 235 bp for forward reads and 142 bp for reverse reads. Following denoising the resulting DADA2-inferred ASVs were subjected to VSEARCH closed-reference clustering against the UNITE dynamic reference database [10] using a single fixed 97% sequence-identity threshold. Sequence clusters obtained after this clustering step are hereafter referred to as operational taxonomic units (OTUs). Taxonomy was then assigned using the QIIME2 feature-classifier classify-sklearn method and the corresponding UNITE reference taxonomy.

In the related workflow, closed-reference clustering was selected to generate a reference-anchored dataset for downstream taxonomic comparison between ITS1, ITS2, and culture-recovered fungi. Consequently, DADA2-inferred ASVs that did not match the selected UNITE reference at the 97% identity threshold were not retained in the downstream OTU table. This implementation therefore prioritizes reference-based taxonomic comparability over retention of all inferred sequence variants and is not intended for de novo novel-taxon discovery.

Use the resulting OTU table, taxonomy table, and sample metadata to compare ITS1 and ITS2 taxonomic profiles and to evaluate their overlap with culture-recovered taxa. Read-count-based interpretation should remain cautious, because PCR cycle number, primer-template mismatches, amplification stochasticity, chimera formation, sequencing depth, reference database coverage, and marker resolution can influence the recovered taxonomic profiles [11]. Therefore, use the ITS1/ITS2 output primarily for taxonomic comparison and method-level complementarity assessment rather than for absolute abundance inference.

#### Comparative taxonomic analysis

Compare the culture-recovered dataset with ITS1 and ITS2 metabarcoding profiles using a taxonomic overlap framework. First, standardize the taxonomic levels across datasets by extracting phylum, class, order, family, genus, and, when appropriate, species-level assignments from the culture-dependent dataset and from the ITS1/ITS2 taxonomy tables. Because species-level assignment from short ITS metabarcoding reads can be uncertain for several fungal groups, prioritize genus and higher taxonomic levels for direct cross-method comparison. Species-level comparisons can be reported descriptively but should be interpreted cautiously, especially for species-rich or taxonomically complex genera.

For presence/absence-based comparative analysis, the ITS1 and ITS2 datasets were restricted to the 14 soil samples for which both culture-dependent and metabarcoding data were available. At each taxonomic level, a taxon was considered detected by metabarcoding when its cumulative read count across these 14 paired samples exceeded one read (>1 read), thereby excluding singleton detections.

Restrict direct culture/metabarcoding overlap analyses to samples for which both culture-dependent and sequence-based data are available. In the related workflow, the comparison between culture-dependent isolation, ITS1 metabarcoding, and ITS2 metabarcoding was therefore performed on the paired sample subset. Generate presence/absence matrices for each method at the selected taxonomic level, typically genus level, and use these matrices to identify taxa that are shared across all approaches, shared by two approaches, or specific to one approach.

Avoid merging colony recovery counts and metabarcoding read counts into a single quantitative abundance estimate, because these outputs are not directly comparable. Culture-derived data reflect fungi that were viable and able to grow under the selected laboratory conditions, whereas ITS read profiles reflect sequence-detected taxa and are influenced by DNA extraction efficiency, primer bias, amplification stochasticity, sequencing depth, and database assignment. For this reason, interpret culture-derived profiles and ITS metabarcoding profiles as method-specific outputs. Use presence/absence-based overlap summaries, Venn diagrams, UpSet plots, or Jaccard similarity to describe methodological complementarity rather than attempting to infer absolute community composition from the combined data.

For visualization, construct bar plots and dot plots to describe the taxonomic composition of each dataset at different taxonomic levels. In the related workflow, GraphPad Prism version 9.1.0 was used to generate bar plots and dot plots for culture-recovered fungi and ITS1/ITS2-assigned OTUs. Venn diagrams comparing taxa recovered by different approaches were generated using InteractiVenn. When phylogenetic representation is useful, construct approximate maximum-likelihood trees using FastTree in MEGA 11 and visualize them with the Interactive Tree of Life (iTOL) platform. These visualizations can be used to show the taxonomic distribution of culture-recovered isolates, the contribution of different culture media, and the overlap between culture-dependent isolation, ITS1 metabarcoding, and ITS2 metabarcoding.

The main outcome of this comparative analysis is the identification of shared and method-specific fungal fractions. Taxa detected by all approaches may represent consistently detectable fungal components within the analysed sample set, whereas culture-only taxa indicate viable fungi recovered under the selected culture conditions but not detected in the ITS profiles. Conversely, ITS-only taxa indicate sequence-detected fungi that were not recovered by culture, either because they were not viable, required growth conditions not represented by the selected media and incubation parameters, were present only as environmental DNA, or did not produce detectable colonies within the seven-day incubation period used in this workflow. This comparative framework is therefore useful for evaluating complementarity between culture-dependent recovery and sequence-based detection, while maintaining a clear distinction between viable fungal resources and metabarcoding-derived taxonomic profiles.

### Workflow application and illustrative results

The workflow was applied in the related research article using soil samples from southeastern France. The culture-dependent workflow yielded 742 purified colonies, corresponding after dereplication to 165 fungal species across 51 genera, 31 families, 18 orders, 9 classes, and 3 phyla. A substantial proportion of recovered species required molecular identification or sequencing confirmation, illustrating the practical value of combining MALDI-TOF MS with targeted multilocus sequencing for environmental fungal isolates.

The five-media design demonstrated medium-dependent recovery. Only 17 of the 165 recovered species were observed on all five media, while 98 species were recovered from a single medium only. FastFung agar yielded the highest number of recovered species and the highest number of medium-specific species in this dataset, supporting the utility of including parallel media for broadening viable fungal recovery in soil fungal isolation workflows. The medium-dependent recovery pattern observed across the five culture media is illustrated in Fig. 4. All uninoculated culture sterility-control plates remained free of fungal growth throughout the incubation period.

**Fig. 4:**
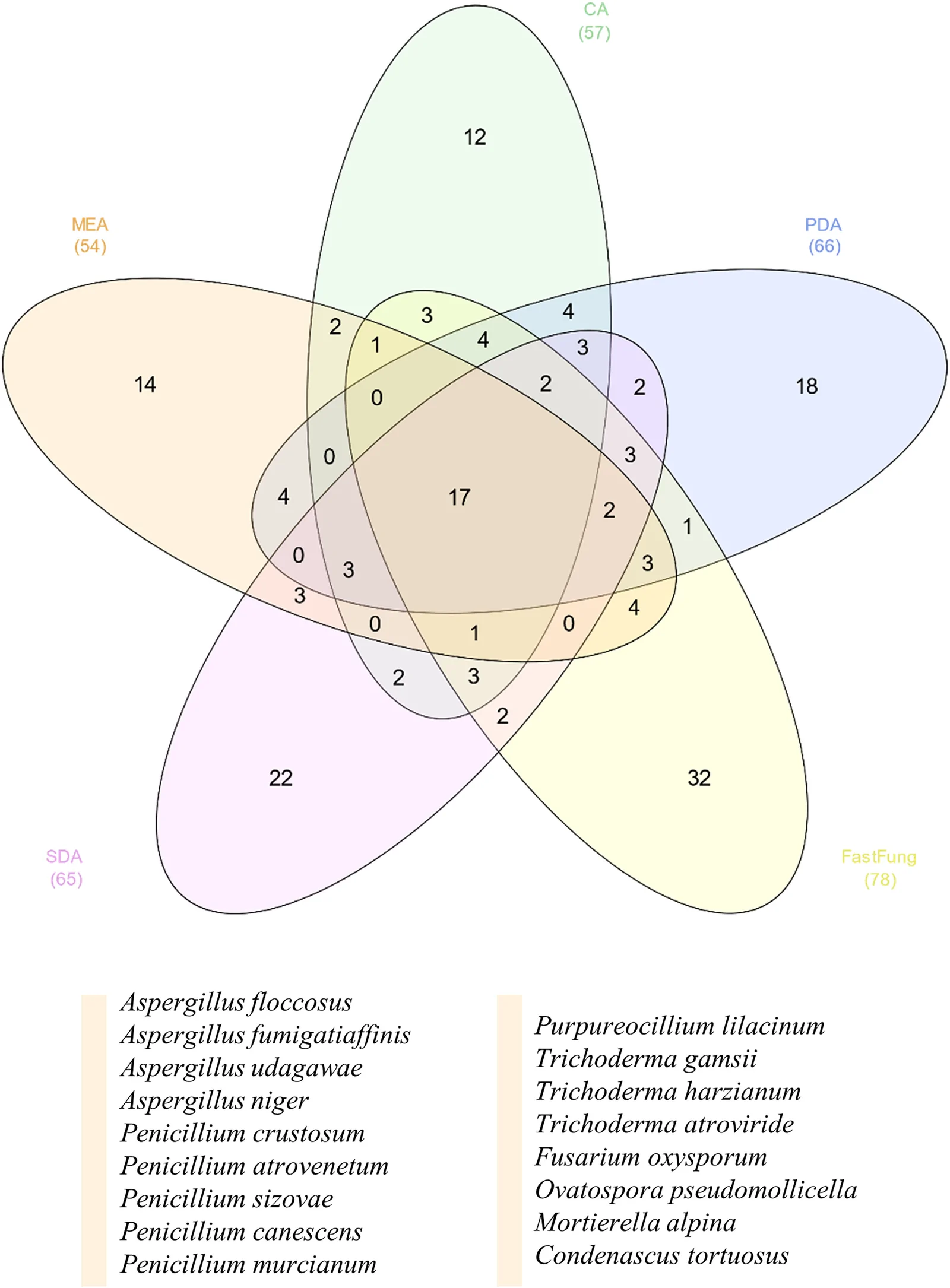
Distribution of culture-recovered fungal species across the five culture media tested. Numbers in parentheses indicate the total number of species recovered on each medium. The central intersection represents the 17 fungal species recovered across all five culture media, which are listed below the Venn diagram. MEA, Malt Extract Agar; CA, CHROMagar Candida; PDA, Potato Dextrose Agar; SDA, Sabouraud Dextrose Agar.

Across the 19 biological soil samples analyzed in the related study, excluding negative controls, the initial sequencing depth ranged from 86,375 to 249,578 reads per sample (mean ≈126,034 reads/sample). Following DADA2 processing, 6814–48,372 non-chimeric reads per sample were retained for ITS1 (mean ≈22,756), whereas 33,025–106,267 reads per sample were retained for ITS2 (mean ≈60,742), corresponding to 432,362 and 1154,095 retained non-chimeric reads, respectively. Metabarcoding negative controls showed only minimal background signal, with no fungal reads detected in the BlancPost controls and only 4 and 6 fungal reads detected in the ITS1 and ITS2 BlancPre controls, respectively.

The ITS1/ITS2 metabarcoding component produced 3235 fungal OTUs, of which 1657 were assigned at least at the phylum level. ITS2 provided a broader assigned taxonomic profile than ITS1 in this dataset, especially for Basidiomycota and Mortierellomycota. Comparison of paired samples showed that culture-dependent isolation and ITS metabarcoding recovered overlapping but distinct fungal fractions, confirming that the workflow can be used to distinguish viable culture-recoverable fungi from sequence-detected taxa. In addition, by identifying taxa repeatedly detected across culture-dependent isolation, ITS1, and ITS2, the workflow can help highlight candidate shared or core-like fungal taxa. When applied to larger datasets covering broader geographical regions, replicated soil types, and more diverse environmental conditions, this approach could therefore contribute to the identification of robust soil-associated fungal components. Figures and supplementary tables supporting these illustrative outcomes are provided in the supplementary material. The taxonomic overlap between culture-dependent isolation, ITS1 metabarcoding, and ITS2 metabarcoding is summarized in Fig. 5.

**Fig. 5:**
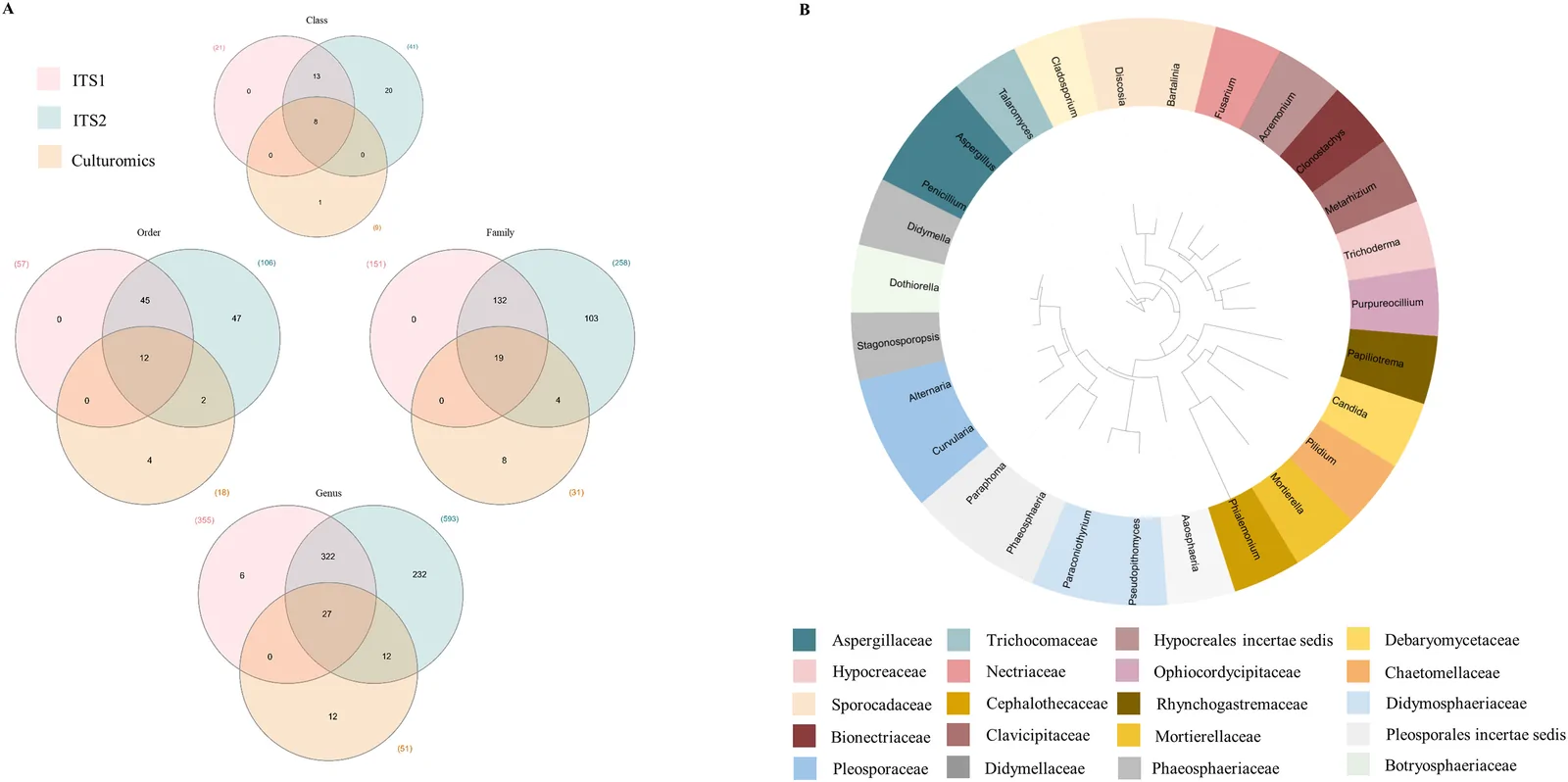
Taxonomic overlap between culture-dependent isolation, ITS1 metabarcoding, and ITS2 metabarcoding. (A) Venn diagrams showing exclusive and shared fungal taxa across the three approaches within the 14 paired samples at the Class (top), Order (lower left), Family (lower right), and Genus (bottom) levels. For ITS1 and ITS2, taxa with a cumulative read count >1 across the paired sample subset were considered present. (B) Phylogenetic distribution of the 27 fungal genera detected by all three approaches within the paired sample subset. Colored ranges indicate fungal families. The approximate maximum-likelihood tree was generated using FastTree and visualized with iTOL.

### Limitations

This workflow is culturomics-inspired but does not reproduce the very large condition matrices used in classical bacterial culturomics. It uses five media, one incubation temperature, aerobic conditions, and a short incubation duration; therefore, it favours fungi that can grow under these selected conditions. Slow-growing, obligately symbiotic, anaerobic, host-dependent, or substrate-specialized fungi may remain unrecovered.

Culture-dependent recovery should not be interpreted as a quantitative estimate of the original soil fungal community. Colony recovery is influenced by spore viability, growth rate, competition, dilution, and medium composition. Similarly, ITS metabarcoding profiles are affected by DNA extraction efficiency, primer-template mismatches, PCR conditions, chimera filtering, clustering strategy, database completeness, and variable ITS resolution among fungal groups. Moreover, because clustering ITS sequences at 97% identity may group closely related fungal taxa, species names returned by downstream taxonomic assignment were treated as taxonomic annotations rather than definitive species delimitations. Although QIIME2 [9] was used in the present workflow, other established bioinformatics platforms, such as mothur [12], may also be used for amplicon-sequencing analysis. The bioinformatics component of this workflow should therefore be considered software-flexible, provided that equivalent processing steps and analytical parameters are clearly documented.

In addition, the closed-reference clustering strategy excludes sequence variants lacking a sufficiently similar reference in the selected UNITE database and may therefore underestimate diversity among poorly represented or novel fungal lineages. Studies specifically targeting sequence-level diversity or novel-taxon discovery should retain DADA2-derived ASVs and/or consider open-reference approaches.

The workflow is best suited for methodological comparison, viable isolate generation, and taxonomic anchoring. It should not be used alone to infer ecological drivers, temporal stability, activity, or full quantitative community structure unless combined with an appropriate replicated sampling design and quantitative molecular approaches.

## Ethics statements

This work did not involve human subjects, animal experiments, or data collected from social media platforms. The study used environmental soil samples only. No specific ethics approval was required for the methodological workflow described here.

## Supplementary material *and/or* additional information [OPTIONAL]

The supplementary files associated with this MethodsX article provide supporting information for reproducing and illustrating the application of the workflow. They include: (i) sample descriptions and primer sets used for isolate sequencing and ITS metabarcoding; (ii) isolate identification tables, including MALDI-TOF MS and molecular identification outputs; (iii) ITS1 and ITS2 taxonomy/read-count tables; (iv) Sanger sequencing and accession information for culture-recovered isolates; (v) summary tables and figures illustrating sequencing output, culture-medium contribution, taxonomic distribution, and overlap between culture-dependent isolation and ITS1/ITS2 metabarcoding; and (vi) pre-clustering DADA2 representative sequences and corresponding feature tables for ITS1 and ITS2, together with sequence variants that did not match the UNITE reference database at the fixed 97% sequence-identity threshold. These supplementary materials are intended to document the datasets underlying the workflow application, enhance transparency and reproducibility, and facilitate reuse, reanalysis, or adaptation of the method in related environmental mycology studies.

## Declaration of competing interest

The authors declare that they have no known competing financial interests or personal relationships that could have appeared to influence the work reported in this paper.

## Data Availability

Suplementary Information.
