# Supplementary material for: A culturomics-inspired multi-medium workflow for soil fungal isolation and ITS metabarcoding comparison
Source: MethodsX. 2026 Sep 27;17:104179. doi: 10.1016/j.mex.2026.104179 (PMC13677964; doi:10.1016/j.mex.2026.104179)
Supplement: Supplementary file 1 [file mmc8.docx]

**Article title: A culturomics-inspired multi-medium workflow for soil fungal isolation and ITS metabarcoding comparison**

**Authors : Charbel Elias; Aïcha Hamieh; Stéphane Ranque; Laure Malleret***

**Affiliations**

**(1) Aix Marseille University, SSA, APHM, RITMES, Marseille, France**

**(2) Aix Marseille University – CNRS, UMR 7376, Laboratory of Environmental Chemistry, Marseille, France**

**(3) IHU Méditerranée Infection, Marseille, France**

**(4) Aix Marseille University – APHM, MEPHI, Marseille, France**

**Corresponding author’s email address and Twitter handle: Laure Malleret: laure.malleret@univ-amu.fr**

**Table of contents**

**Supplementary Table 1.** Sample identifiers and available source, site, sampling, and collection-date metadata for the 19 soil samples analyzed in this study.

**Supplementary Table 2.** Sets of primers employed for the amplification of the ITS, B-tub2, TEF1, LSU (28S), and RPB2 genetic regions.

**Supplementary Table 3.** Overview of the ITS1/ITS2 amplicon-based Illumina sequencing data.

**Supplementary Table 4.** Taxonomic distribution of fungal isolates recovered by culturomics and OTUs detected by ITS1/ITS2 metabarcoding. ITS1 and ITS2 taxon totals are shown for the complete 19-sample metabarcoding dataset using cumulative read-count thresholds of >1, >10, and >100 reads.

**Supplementary Table 1.** Sample identifiers and available source, site, sampling, and collection-date metadata for the 19 soil samples analyzed in this study.

| **Sample ID** | **Source / provider** | **Site or sample label** | **Additional sampling information** | **Collection date** |
| --- | --- | --- | --- | --- |
| SS1 | Environmental association | Port-Saint-Louis-du-Rhône | Anthropized soil | 29/11/2020 |
| SS2 | SNCF | Railroad track | Anthropized soil | 22/08/2017 |
| SS3 | French Tech in nature-based solutions | Train station Auvergne-Rhône-Alpes region | Far from human activities | 04/2022 |
| SS4 | French Tech in nature-based solutions | Train station Auvergne-Rhône-Alpes region” | Near the tracks | 04/2022 |
| SS5 | French Tech in nature-based solutions | Train station Auvergne-Rhône-Alpes region | Far from human activities sifted and freeze-dried | 04/2022 |
| SS6 | French Tech in nature-based solutions | Train station Auvergne-Rhône-Alpes region | Near the tracks sifted and freeze-dried | 04/2022 |
| SS7 | Environmental association | Port-Saint-Louis-du-Rhône (ref S5) | Unsorted soils | 11/03/2020 |
| SS8 | Environmental association | Port-Saint-Louis-du-Rhône (ref S9) | Topsoil | 11/03/2020 |
| SS9 | Environmental association | Port-Saint-Louis-du-Rhône (ref S1) | Industrial wasteland | 05/03/2020 |
| SS10 | Environmental association | Port-Saint-Louis-du-Rhône (ref S2) | Topsoil | 05/03/2020 |
| SS11 | Environmental association | Port-Saint-Louis-du-Rhône | Unsorted soils | 11/03/2020 |
| SS12 | BioCentre | Industrial soil | Polluted soil | 06/2023 |
| SS13 | BioCentre | Industrial soil | Polluted soil | 06/2023 |
| SS14 | BioCentre | Industrial soil | Polluted soil | 06/2023 |
| SS15 | BioCentre | Industrial soil | Polluted soil | 06/2023 |
| SS16 | BioCentre | Industrial soil | Polluted soil | 06/2023 |
| SS17C | Environmental Chemistry Laboratory | Topsoil Technopole in Aix-en-Provence | Sifted | 12/2023 |
| SS18C | Environmental Chemistry Laboratory | Private garden in La Verdière | Sifted | 12/2023 |
| SS19C | Environmental Chemistry Laboratory | Municipal garden in Les Pennes-Mirabeau | Sifted | 12/2023 |

**Supplementary Table 2.** Sets of primers employed for the amplification of the ITS, B-tub2, TEF1, LSU (28S), and RPB2 genetic regions.

| **Primers** |  | **Sequences** |  | **Targeted Regions** |  |
| --- | --- | --- | --- | --- | --- |
| ITS1 | TCCGTAGGTGAACCTGCGG | | | 18S-5.8S |  |
| ITS2 | GCTGCGTTCTTCATCGATGC | | | 18S-5.8S |  |
| ITS3 | GCATCGATGAAGAACGCAGC | | | 5.8S-28S |  |
| ITS4 | TCCTCCGCTTATTGATATGC | | | 5.8S-28S |  |
| ITS1 | TCCGTAGGTGAACCTGCGG | | | 18S-5.8S, 5.8S-28S |  |
| ITS4 | TCCTCCGCTTATTGATATGC | | | 18S-5.8S, 5.8S-28S |  |
| Bt-2a | CATCGAGAAGTTCGAGAAGG | | | B-tub2 |  |
| Bt-2b | TACTTGAAGGAACCCTTACC | | | B-tub2 |  |
| EF1-728F | GGTAACCAAATCGGTGCTGCTTTC | | | TEF1 |  |
| EF1-986R | ACCCTCAGTGTAGTGACCCTTGGC | | | TEF1 |  |
| D1 | AACTTAAGCATATCAATAAGCGGAGGA | | | 28S |  |
| D2 | GGTCCGTGTTTCAAGACGG | | | 28S |  |
| fRPB2-5F | GAYGAYMGWGATCAYTTYGG | | | RNA polymerase II gene |  |
| fRPB2-7cr | CCCATRGCTTGTYYRCCCAT | | | RNA polymerase II gene |  |
| EF1 (*Trichoderma*) | ATGGGTAAGGARGACAAGAC | | | TEF1 |  |
| EF2 (*Trichoderma*) | GGARGTACCAGTSATCATGTT | | | TEF1 |  |

**Supplementary Table 3.** Overview of the ITS1/ITS2 amplicon-based Illumina sequencing data.

|  |  | Raw reads (Combined)^1^ |  |  |  | Clean reads^2^ |  |  | OTUs^3^ | |
| --- | --- | --- | --- | --- | --- | --- | --- | --- | --- | --- |
|  | Total | Minimum | Maximum |  | Reads after quality filtering and denoising | Merged reads | Non-chimeric reads |  | Total | The number of assigned OTUs |
|  |  |  |  |  |  |  |  |  |  |  |
| ITS1 | 2,394,642 | 86,375 | 249,578 |  | 926,894 | 584,164 | 432,362 |  | 1040 | 595 |
| ITS2 |  |  |  |  | 1,888,227 | 1,338,600 | 1,154,095 |  | 2195 | 1062 |

**Supplementary Table 4.** Taxonomic distribution of fungal isolates recovered by culturomics and OTUs detected by ITS1/ITS2 metabarcoding. ITS1 and ITS2 taxon totals are shown for the complete 19-sample metabarcoding dataset using cumulative read-count thresholds of >1, >10, and >100 reads.

|  |  | ITS1 |  |  |  | ITS2 |  |  | Culturomics |
| --- | --- | --- | --- | --- | --- | --- | --- | --- | --- |
|  | Total ( >1 read) | Total ( >10 reads) | Total ( >100 reads) |  | Total ( >1 read) | Total ( >10 reads) | Total ( >100 reads) |  | Total |
| Phylum | 8 | 5 | 4 |  | 12 | 11 | 7 |  | 3 |
| Class | 23 | 19 | 14 |  | 45 | 37 | 25 |  | 9 |
| Order | 63 | 53 | 35 |  | 116 | 96 | 61 |  | 18 |
| Family | 164 | 143 | 92 |  | 282 | 229 | 142 |  | 31 |
| Genus | 394 | 341 | 183 |  | 650 | 530 | 303 |  | 51 |
| Species | 563 | 486 | 244 |  | 1,062 | 861 | 439 |  | 165 |
